# What factors at discharge predict physical activity and walking outcomes 6 months after stroke? A systematic review

**DOI:** 10.1177/02692155241261698

**Published:** 2024-07-25

**Authors:** Neelam Nayak, Niruthikha Mahendran, Suzanne Kuys, Sandra G Brauer

**Affiliations:** 1 Physiotherapy, School of Health and Rehabilitation Sciences, 1974 University of Queensland, Brisbane, Australia; 2 Physiotherapy, School of Allied Health, Faculty of Health Sciences, Australian Catholic University, Brisbane, Australia

**Keywords:** Stroke, physical activity, walking outcomes, predictors, discharge‌

## Abstract

**Objective:**

This study aimed to identify factors at hospital discharge that predict physical activity and walking outcomes in the first 6 months after stroke.

**Data sources:**

Searches were conducted in CINAHL (EBSCO), Web of Science, PubMed and Scopus from inception to 30 April 2024. Reference lists of included articles were manually screened to identify additional studies.

**Review methods:**

Studies of adults with stroke reporting predictors at hospital discharge and outcomes of physical activity or walking across the first 6 months after hospital discharge were included. Two reviewers independently screened titles, abstracts and reviewed full texts. Quality of included studies was assessed with Quality in Prognostic Studies screening tool. A narrative synthesis was undertaken.

**Results:**

The search strategy retrieved 7834 studies, from which 6 eligible studies were identified, including a total of 1433 participants. Overall, studies had a low risk of bias. Age, balance, walking speed and walking distance at hospital discharge predicted physical activity outcomes after stroke (*n* = 2 studies). Cognition, lower limb cycling rhythm and self-efficacy for walking at hospital discharge predicted walking outcomes after stroke (*n* = 4 studies).

**Conclusions:**

A range of factors predicted physical activity and walking outcomes 6 months after stroke. Physical capabilities at discharge appear to be a predictor of these outcomes; however, this needs to be interpreted with caution. Diverse measures and time points were used across studies to characterise physical activity and walking outcomes, highlighting the need for consistency in measurement and longitudinal studies in stroke research.

Stroke is one of the leading causes of disability worldwide and the second most common cause of global disability-adjusted life years (DALYs).^1^ Most individuals with stroke remain physically inactive and spend most of their day in sedentary behaviours.^2,3^ This increases their risk of recurrent stroke^4,5^ and poor health outcomes.^6^ Further, walking recovery, a common approach to increasing physical activity, is suboptimal post-stroke.^7^ Most individuals with stroke continue to report limited walking and poor satisfaction with walking outcomes.^7^ Thus, targeted interventions aimed at improving and maintaining physical activity and walking after stroke are necessary to reduce burden and improve long-term health outcomes.^8,9^ Understanding factors amenable to intervention is essential for designing successful interventions.

Hospital discharge is a critical juncture in rehabilitation after stroke. Understanding factors at hospital discharge predictive of physical activity and walking in the months following is crucial for several reasons. First, predictors identified around hospital discharge could drive decisions regarding both the service and content of rehabilitation post-discharge. Second, as physical activity and walking ability are integral for transitioning to home and community, understanding important factors at discharge could influence decisions regarding discharge destination. Lastly, if likely predictors of activity and walking ability are known at discharge, future inpatient intervention approaches could focus on optimising these factors. The subacute phase is a key time period where neurological recovery potential is high.^10,11^ Targeted interventions in a supportive rehabilitation environment could further minimise physical inactivity behaviour that often occurs in later stages when individuals with stroke transition home after discharge.

To date, a 2018 systematic review^12^ found that after stroke, factors including cardiorespiratory fitness, fatigue, falls and balance self-efficacy were significantly associated with physical activity. However, the majority of studies were cross sectional and included participants in the chronic phase of stroke.^12^ Another systematic review^13^ investigated predictors of independent walking across the subacute phase of stroke and found a variety of factors including age, an intact corticospinal tract, cognition, sitting balance and continence early post-stroke onset.^13^ This review, however, included only independent walking as an outcome and restricted inclusion to non-ambulatory individuals with stroke.^13^ Thus, it is not clear what factors at hospital discharge prospectively predict physical activity levels, or walking independence, speed or distance after stroke, as they transition home and recover. This systematic review aims to determine the following: (1) What factors at hospital discharge predict physical activity outcomes within first 6 months after stroke? and (2) What factors at hospital discharge predict walking outcomes within first 6 months after stroke?

## Methods

This systematic review followed the Preferred Reporting Items for Systematic Reviews and Meta-analysis (PRISMA) guidelines^14^ and was prospectively registered with the International Prospective Register of Systematic Reviews (CRD42020202323).

Inclusion and exclusion criteria were determined a priori. Studies of human participants with first or recurrent stroke, aged 18 years or above, were included. Primary research studies consisting of prospective cohort studies, randomised trials and prognostic and predictive studies, reporting on outcomes of physical activity or walking across the first 6 months after stroke and exploring independent factors around the time of hospital discharge were included. Studies that provided univariate or multivariate statistics of prediction between the independent factors and physical activity or walking outcomes were included. Studies reporting outcomes of physical activity, such as accelerometer-derived number of steps, frequency, intensity, duration of physical activity^15^ and physical activity diaries or questionnaires, or walking recovery outcomes such as walking independence, speed and distance^7^ were included. Studies not in English were excluded.

Electronic search strategies were developed in consultation with a research librarian and included keywords relevant to physical activity and walking after stroke (Supplemental material S1). Search strategies were used across the bibliographic databases of CINAHL (EBSCO), Web of Science, PubMed and Scopus from inception to 30 April 2024. Where relevant, Medical Subject Headings and indexed terms were used to provide specific subject headings for each database. The search was limited to the English language and to human studies. The reproducible searches for all databases are available in the Supplemental material. Citation searches of included studies were undertaken in Web of Science. Reference lists of included studies were screened to identify any further relevant studies. For each database, auto alerts were set up to provide monthly updates of new literature until 30 April 2024.

The yield from database searches was uploaded to the web-based collaboration software platform, Covidence (Available at www.covidence.org). Duplicates were removed by using EndNote’s duplicate identification strategy. All titles and abstracts were screened against the eligibility criteria independently by two reviewers (two of NN, NM, SB and SK). In cases where it was not clear if eligibility criteria had been met or reviewers did not agree, papers were retained for full-text review. Full-text copies were reviewed independently by two reviewers (NN and SK). Any disagreement was resolved with a third reviewer (NM and SB).

The quality of included studies was assessed with the Quality in Prognostic Studies (QUIPS) screening tool for prognostic factor studies.^16^ The screening tool consists of six domains of bias for prognostic factors: study participation, study attrition, prognostic factor measurement, confounding measurement, outcome measurement and analysis and reporting. Studies were rated as having low, moderate or high risk of bias in relation to each of these domains.^16^ Included studies were assessed for quality independently by two reviewers (NN and NM) with disagreements resolved by a third reviewer (SK).

Data were extracted by one investigator (NN), stored in Covidence and independently cross-checked by another reviewer (SK and NM). Data were cross-checked for author, study and journal details, including date, title and details of publication, and participant data related to age and sex, total number of participants in the study and time after stroke. Information related to physical activity and walking outcomes (e.g., measurement and time point), and statistical association details were extracted and checked.

Descriptive data synthesis was undertaken based on participant characteristics and identified factors at discharge related to physical activity and walking outcomes after stroke and the association/correlational statistics. A meta-analysis of the pooled estimates between identified factors and outcomes of physical activity and walking recovery was planned but was not possible due to heterogeneity across studies for reported predictors and physical activity and walking recovery outcomes. Thus, a narrative synthesis was conducted.

## Results

**Figure 1.**
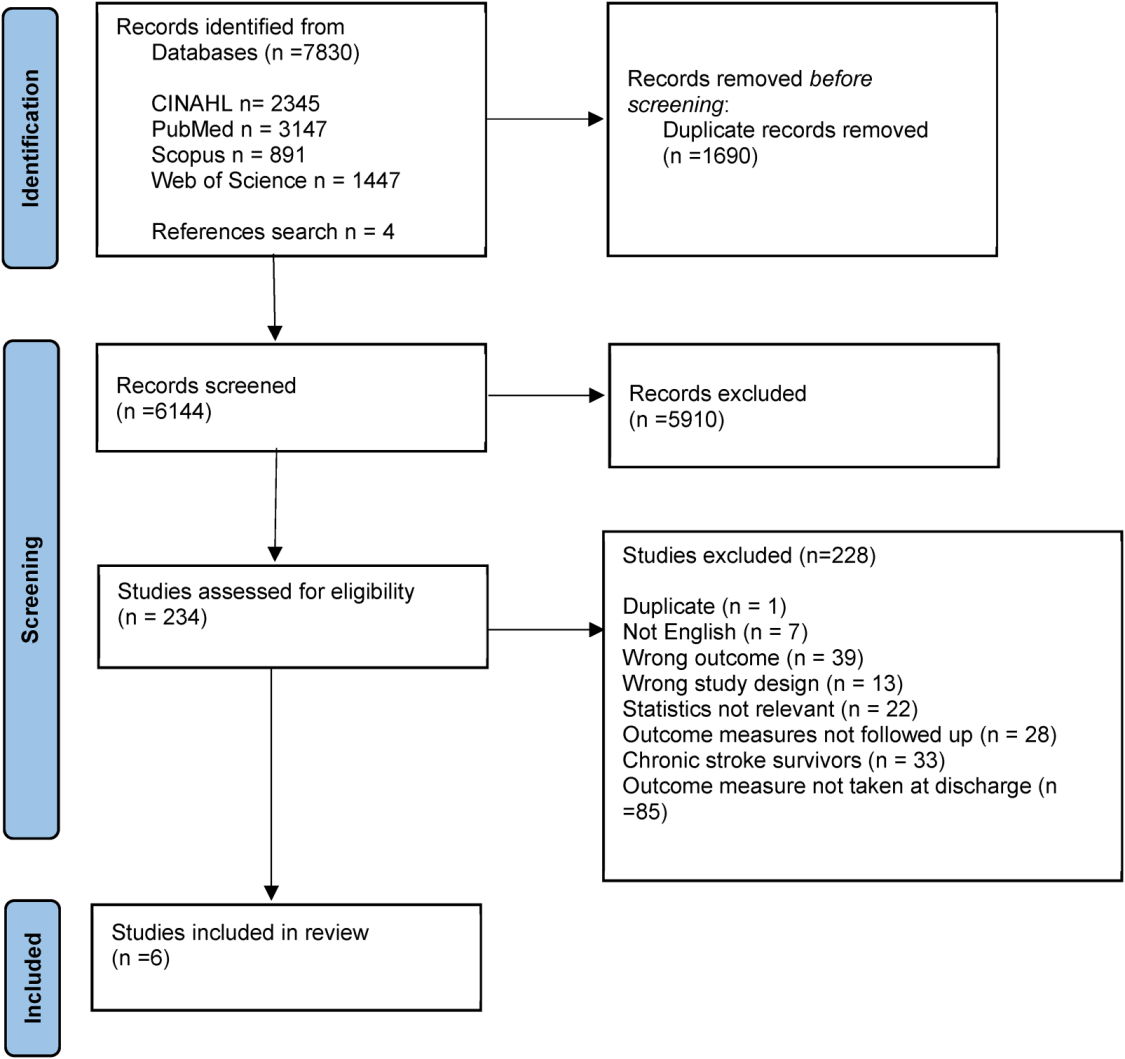
PRISMA flow chart of systematic review search process.

A total of 7830 citations were retrieved from the 4 databases and 4 identified from reference list searches, thus yielding a total of 7834 studies. After removing duplicates, 6144 studies were screened for titles and abstracts. Of these, 234 studies were retrieved as full text. Following full-text review, six studies^17–22^ were included in final synthesis (Figure 1) and are summarised in Table 1.

**Table 1.** Summary of the included studies.

Study	Participants	Predictors investigated	Outcomes of physical activity or walking	Time points for measurement	Factors predicting outcomes
*Studies reporting on physical activity outcomes*					
Mahendran 2020^19^ (Australia)	*n* = 36	Age Pre-stroke activity (PASE) Fatigue (FSS-7) Mood (HDS) Executive function (TMTA and B) Walking speed (10MWT) Walking distance (6MWT) Walking self-efficacy (ASCQ) Perceived stroke recovery (SIS) Perceived health (EQ-5D)	Daily step count	Predictors: At discharge from acute stroke unit and inpatient rehabilitation	**All physical activity outcomes at *1 month*:** Walking distance alone (adj *R*^2 ^= 0.26–0.44, *p* < 0.005)
			Frequency of PA (time in long activity bouts)		
	Age (*y*) = 71 (14)				
					**Daily step count and intensity of physical activity at *3 months*:** Walking distance and pre-stroke activity (adj *R*^2 ^= 0.41–0.58, *p* < 0.007)
	Sex = 11 F, 25 M		Intensity of PA (time in high intensity bouts) (accelerometer-derived)		
				Outcomes: 1, 3 and 6 months after discharge	
	Time after stroke (*days*) = 24 (21)				**Frequency of physical activity at *3 months*:** pre-stroke activity levels alone (adj *R*^2 ^= 0.28, *p* = 0.004)
					**Daily step count and frequency of physical activity at *6 months*:** Age alone (adj *R*^2 ^= 0.31–0.32, *p* < 0.003)
					**Intensity of physical activity at *6 months*:** Walking distance, pre-stroke activity and executive function (adj *R*^2 ^= 0.76, *p* < 0.001)
Thilarajah 2020^22^ (Singapore)	*n* = 55	Comfortable walking speed (10-minWT) Step test Ankle dorsiflexor strength Cognition (MoCA) Anxiety and depression (HADS) Falls self-efficacy (FES-I) Motivation (BREQ-2)	Daily step count (accelerometer-derived) self-reported intensity of PA (IPAQ-S7)	Predictors: Within 1-week prior to discharge from inpatient rehabilitation	**Daily step count:** Walking speed (*p* = 0.04), step test with affected (*p* = 0.03) and unaffected limb (*p* < 0.01)
	Age (*y*) = 59 (49–67)				
	Sex = 29 F, 35 M				**Self-reported physical activity intensity:** Dorsiflexor strength in affected limb (*p* < 0.01) and step test with unaffected limb (*p* = 0.02)
				Outcomes: 3 months after discharge	
			Self-reported participation in PA (ACS)		
	Time after stroke (*days*) = median 24 (IQR 20 to 36)				
					**Self-reported participation in physical activity:** Walking speed (*p* < 0.01), step test with unaffected limb (*p* < 0.01), step test with affected limb (*p* < 0.001), anxiety (*p* = 0.03) and motivation (*p* = 0.04).
*Studies reporting on walking outcomes*					
Bonetti 2008^17^ (UK)	*n* = 203	Locus of control Walking self-efficacy Perceived behavioural control Walking intention Neurological impairment	Walking limitation (functional limitation profile: ambulation subscale)	Predictors: Within 2 weeks after discharge from acute stroke unit.	**Walking limitation:** Perceived behavioural control = adj *R*^2 ^= 0.19, *p* < 0.001) and walking recovery
	Age (*y*) = 69 (12)				
			Walking recovery (walking limitation score)	Outcomes: 6 months after discharge	**Walking recovery:** Perceived behavioural control (adj *R*^2 ^= 0.11, *p* < 0.001)
	Sex = 79 F, 124 M				
	Time after stroke = Not reported				
Katz-Leurer 2005^18^ (Israel)	*n* = 44	Lower limb cycling: cycling at 50 r/min with and without 10 W resistance for 1 min Lower limb strength (SSS) Walking limitation	Walking speed (10MWT)	Predictors: 15 days post stroke	**Walking speed:** Ability to cycle at 50 r/min with and without 10 W resistance (*R*^2 ^= 0.40, *p* < 0.01)
			Walking distance until fatigue		
	Age (*y*) = 65 (11)		Number of stairs climbed until fatigue	Outcomes: 3 months after stroke	
					**Walking distance:** Ability to cycle at 50 r/min against 10 W resistance (*R*^2 ^= 0.21, *p* < 0.01)
	Sex =22 F, 22 M				
	Time after stroke *(days) *= 15				**Number of stairs climbed:** Ability to cycle at 50 r/min with and without 10 W resistance (*R*^2 ^= 0.26, *p* < 0.01)
Nakao 2020^20^ (Japan)	*n* = 1023	Age Sex Stroke type Length of hospital stay FIM cognition and motor score Falls self-efficacy (FES) Hemiparesis severity (Brunnstrom) Time Up and Go (TUG) Walking distance > 100 m Social Network Scale	Walking participation via life–space assessment (LSA)	Predictors: At discharge from inpatient rehabilitation	**Walking participation (LSA):** Female sex, age, falls efficacy FIM cognitive score and walking speed and length of stay together (R^2 ^= 0.54, *p* < 0.05)
	Age (*y*) = 65 (58–73)				
	Sex = 347 F, 676 M			Outcomes: 2 months after discharge	
	Time after stroke (*days*) = median 97 (IQR 66–134)				
Park 2017^21^ (Korea)	*n* = 72	Age Sex Stroke severity (NIHSS) Stroke side Lower limb strength (FM and Knee extensor power) Cognitive function (WMT, VFT, BNT, CPT)	Walking independence (FAC)	Predictors: Within 2 weeks of discharge from inpatient rehabilitation	**Walking independence:** Verbal fluency (*R*^2 ^= 0.454), word list memory (*R*^2 ^= 0.449), construction praxis (*R*^2 ^= 0.433) and Boston naming test (*R*^2 ^= 0.424), when adjusted for age, lower limb strength and side of stroke
			Walking participation via ambulatory zones (household versus community ambulator)		
	Age (*y*) = 69 (11)				
	Sex = 41 F, 31 M			Outcomes: 6 months after stroke	
	Time after stroke (*days*) = 27 (12)				**Walking participation:** Word list memory (*R*^2 ^= 0.613), verbal fluency test (*R*^2 ^= 0.506), construction praxis (*R*^2 ^= 0.497) and Boston naming test (*R*^2 ^= 0.413) when adjusted for age, lower limb strength and side of stroke

All data is presented as mean (SD) unless otherwise specified.

r/min: repetitions per minute; SSS: Scandinavian Stroke Scale; 10MWT:  10-metre walk test; PASE: physical activity scale for the elderly; FSS-7: Fatigue Severity Scale (7-item); HDS: Happiness and Depression Scale; TMT-A: Trail Making Test Part A; TMT-B: Trail Making Test Part B; 6MWT: 6-minute walk test; ASCQ: Ambulatory Self-Confidence Questionnaire; SIS: Stroke Impact Scale; EQ-5D: European Quality of Life Instrument; FIM: Functional Independence Measure; FES: Falls Efficacy Scale; TUG: Timed Up and Go test; LSA: life–space assessment; NIHSS: National Institutes of Health Stroke Scale; FM: Fugl–Meyer Scale; WMT: word list memory test; VFT: verbal fluence test; BNT: Boston naming test; CPT: construction praxis test; FES-I: Falls Efficacy Scale (International); FAC: functional ambulation categories; 10-minWT: 10-minute walk test; MoCA: Montreal cognitive assessment; HADS: Hospital Anxiety and Depression Scale; BREQ-2: Behavioural Regulation in Exercise Questionnaire-2; ACS: activity card sort – high demand leisure; IPAQ-S7: International Physical Activity Questionnaire-Short 7 Days.

Studies were published between 2005 and 2020 and included a total of 1433 participants (study samples ranging from *n* = 36 to 1023). Four studies reported outcome measures related to walking and two studies reported outcomes related to physical activity. All studies were longitudinal observational cohort studies, presenting multivariate statistics of association.

All studies had an overall low risk of bias (Table 2). All studies had a low risk of selection bias. Four (67%) studies had a low^17–19,22^ and two (33%) had a moderate^20,21^ risk of attrition bias. All studies had a low risk of measurement bias in relation to the prognostic/predictive factors and measurement bias in relation to the outcome. Four (67%) studies had a moderate risk of bias due to confounding,^19–22^ and two (33%) had a high risk.^17,18^

**Table 2.** Quality of the included studies (*n* = 6).

Study	Selection bias	Study attrition	Prognostic factor measurement	Outcome measurement	Study confounding	Statistical analysis and reporting	Overall risk
Bonetti 2008^17^	Low	Low	Low	Low	High	Low	Low
Katz-Leurer 2005^18^	Low	Low	Low	Low	High	Low	Low
Mahendran 2020^19^	Low	Low	Low	Low	Moderate	Low	Low
Nakao 2020^20^	Low	Moderate	Low	Low	Moderate	Low	Low
Park 2017^21^	Low	Moderate	Low	Low	Moderate	Low	Low
Thilarajah 2020^22^	Low	Low	Low	Low	Moderate	Low	Low

Two studies (*n* = 91) investigated factors at hospital discharge able to predict physical activity outcomes at 1,^19^ 3^1^^9,22^ and 6 months^19^ after discharge (Table 1). Both studies reported daily step counts, measured with different accelerometers, the ActivPAL^TM 19^ and a custom ankle-mounted accelerometer.^22^ In addition, accelerometer-derived time in high intensity (intensity) and long duration (frequency) walking bouts^19^ and self-reported intensity and participation in physical activity^22^ was reported.

Walking distance (6-minute walk test (6MWT)) at hospital discharge predicted daily step count, intensity and frequency of physical at 1 month after discharge.^19^ Comfortable walking speed,^22^ step test performance,^22^ ankle dorsiflexor strength^22^ and walking distance and pre-stroke physical activity^19^ predicted physical activity 3 months after hospital discharge. Age, walking distance, pre-stroke physical activity^19^ and executive function^19^ at hospital discharge predicted physical activity 6 months later.

Four studies (*n* = 1342) investigated factors at discharge associated with walking outcomes at 2,^20^ 3^18^ and 6 months^17,21^ after discharge (Table 1). Each study reported a different measure of walking, including clinical outcomes of walking independence,^21^ walking speed and walking distance,^18^ self-reported outcomes of walking participation,^21^ life–space assessment^20^ and perceived walking limitation and recovery.^17^

Walking independence at 6 months after discharge was predicted by cognitive function, when adjusted for age, lower limb strength and side of stroke (*R*^2 ^= 0.424 to 0.454).^21^ Walking speed at 3 months after hospital discharge was predicted by the ability to cycle at a constant rate with and without resistance at discharge (*R*^2 ^= 0.40),^18^ while walking distance and the ability to climb stairs at 3 months after hospital discharge were predicted by the ability to cycle at a constant rate with resistance only (*R*^2 ^= 0.21 and 0.26, respectively).^18^

Self-reported walking participation at 2 months after discharge^20^ was predicted by female sex, age, falls self-efficacy, cognitive function, walking speed (via Tmed Up and Go test) and length of hospital stay collected at hospital discharge (*R*^2 ^= 0.54). Walking participation at 6 months^21^ was predicted by cognitive function when adjusted for age, lower limb strength and side of stroke (*R*^2 ^= 0.497 to 0.613).^21^ Self-reported perceived walking limitation and recovery at 6 months were predicted by perceived behavioural control only (adjusted *R*^2 ^= 0.19 and 0.11, respectively).^17^

## Discussion

This systematic review identified 6 studies (*n* = 1433, low risk of bias) that investigated factors at hospital discharge predicting physical activity (2 studies) and walking outcomes (4 studies) during the first 6 months after stroke. Physical capabilities at discharge appear to be a predictor of these outcomes; however, this needs to be interpreted with caution. Diverse measures and time points were used across studies to characterise physical activity and walking outcomes across the subacute phase of stroke, preventing meta-analysis. Physical activity and walking outcomes identified in this review represent distinct clinical targets in post-stroke rehabilitation. Walking independence,^28,29^ speed^23,30,31^ and distance^23,29,30^ capture different targets of recovery^32^ and relate to varied participation goals for people living with stroke.^24,33^ Consequently, the diverse predictors found in this review are unsurprising. Nevertheless, this review highlights the need for consistency in outcome measurement and for longitudinal studies in stroke research.

This review found that most studies that measured physical or walking capability at hospital discharge determined that it significantly predicted physical activity or walking outcomes up to 6 months later but only explained a part of the outcome. While it is likely that higher physical capability will enable improvements in physical activity and walking, other factors will need consideration and intervention when aiming to increase *long-term* physical activity and walking outcomes after stroke.^25^ This is supported by the finding that various measures of cognitive function often contributed to the prediction equations in the examined studies in this review. This finding supports previous studies that have identified that factors important in behaviour change contribute to recovery of physical activity and walking post-stroke.^26,27,34,35^ This supports the premise that to successfully enhance long-term physical activity and walking recovery, rehabilitation should include strategies such as behaviour change techniques.^9,36,37^ Clearly, further investigation is required to understand what techniques to select, for whom, when and how to optimally use these techniques to enhance recovery of physical activity and walking as individuals with stroke transition home.

Previous research has identified factors on hospital admission such as age^38–41^ and stroke severity^39,42,43^ that predict physical activity 6 months after stroke. In contrast, this systematic review included papers that identified modifiable predictors at a later time point which could be targeted with tailored intervention during rehabilitation. Understanding what modifiable factors predict positive physical activity and walking outcomes can help clinicians focus on factors to optimise prior to discharge, set realistic goals and foresee needs for additional care after discharge. In addition, it is of clinical importance to identify individuals with stroke at risk of becoming inactive as they transition home. This is a key step required to enable tailored and proactive strategies to improve long-term physical activity and walking outcomes after stroke.

Diverse outcomes of physical activity and walking identified in this review call for a need for consistency in measures used in post-stroke rehabilitation and research. A recent international consensus on measuring physical activity recommends using device-based measures for step count.^15^ Likewise, the Stroke Recovery and Rehabilitation Roundtable (SRRR)^44^ recommendes the use of standardised measurement for walking outcomes, including tests such as 10-metre walk test for walking speed. Implementing a standardised protocol for collecting data, such as wear time of devices, walkway length for tests and frequency of data collection, may be useful to ensure consistency across different studies. Consistent measurement protocols would enable pooling data from multiple studies, facilitating meta-analyses.

There was also a lack of consistency in the time points used for follow-up by studies included in this review, ranging from 4 weeks to 6 months after stroke, making it difficult to draw conclusions. Measurements of physical activity and walking outcomes at fixed time points,^45^ across the subacute stage after stroke,^46^ are recommended. Consensus from SRRR^45^ recommends that measurement of outcomes should commence within first 7 days of stroke and occur at designated intervals up to at least 3 months post-stroke. Standardising fixed time points could enable longitudinal analyses of data, thereby facilitating identification of predictors of recovery after stroke and intervention effectiveness.

Limitations of this review must be acknowledged. The review found only 6 studies that met inclusion criteria, with participant numbers ranging from *n* = 36 to 1023, which highlights the need for prospective longitudinal studies to explore factors associated with physical activity and walking after stroke. Positively, the studies had low risk of bias and used multivariate statistics. However, there was heterogeneity of outcome measures and time points in the included studies, which limited synthesis via a meta-analysis, highlighting the need for consistency in measurement in future research.

This review found a range of factors related to physical activity and walking outcomes across the first 6 months following hospital discharge, with physical capability as the most frequently identified predictive factor. More larger longitudinal studies with a breadth of predictor variables and standardised outcome measures at agreed time points are needed to inform the design of targeted interventions to improve physical activity and walking recovery after stroke.

**Clinical messages**

- Physical capability at hospital discharge contributes to prediction of physical activity or walking outcomes across the first 6 months of returning home after stroke.
- Different factors at hospital discharge predict the diverse measures used to characterise physical activity and walking outcomes across the subacute phase post-stroke.
- Consistency in measurement of physical activity and walking after stroke is needed.

## Supplemental Material

sj-docx-1-cre-10.1177_02692155241261698 - Supplemental material for What factors at discharge predict physical activity and walking outcomes 6 months after stroke? A systematic review Supplemental material, sj-docx-1-cre-10.1177_02692155241261698 for What factors at discharge predict physical activity and walking outcomes 6 months after stroke? A systematic review by Neelam Nayak, Niruthikha Mahendran, Suzanne Kuys and Sandra G Brauer in Clinical Rehabilitation

sj-docx-2-cre-10.1177_02692155241261698 - Supplemental material for What factors at discharge predict physical activity and walking outcomes 6 months after stroke? A systematic review Supplemental material, sj-docx-2-cre-10.1177_02692155241261698 for What factors at discharge predict physical activity and walking outcomes 6 months after stroke? A systematic review by Neelam Nayak, Niruthikha Mahendran, Suzanne Kuys and Sandra G Brauer in Clinical Rehabilitation

## References

[1] Tsao CW, Aday AW, Almarzooq ZI, et al. Heart disease and stroke statistics—2022 update: a report from the American Heart Association. Circulation 2022; 145: e153–e639. 10.1161/CIR.0000000000001052 35078371

[2] English C Healy GN Coates A , et al. Sitting and activity time in people with stroke. Phys Ther 2016; 96: 193–201. 26112254 10.2522/ptj.20140522

[3] Fini NA Bernhardt J Holland AE . Low gait speed is associated with low physical activity and high sedentary time following stroke. Disabil Rehabil 2021; 43: 2001–2008. DOI: 10.1080/09638288.2019.1691273 31755311

[4] Turan TN Nizam A Lynn MJ , et al. Relationship between risk factor control and vascular events in the SAMMPRIS trial. Neurology 2017; 88: 379–385. 28003500 10.1212/WNL.0000000000003534 PMC5272964

[5] Elnady HM Mohammed GF Elhewag HK , et al. Risk factors for early and late recurrent ischemic strokes. Egypt J Neurol Psychiatr Neurosurg 2020; 56: –7.

[6] Al-Fayyadh S . Predicting the functional independence during the recovery phase for poststroke patients. Nurs Open 2019; 6: 1346–1353. 31660161 10.1002/nop2.335 PMC6805273

[7] Moore SA Boyne P Fulk G , et al. Walk the talk: current evidence for walking recovery after stroke, future pathways and a mission for research and clinical practice. Stroke 2022; 53: 3494–3505. 36069185 10.1161/STROKEAHA.122.038956 PMC9613533

[8] Moore SA Hrisos N Flynn D , et al. How should long-term free-living physical activity be targeted after stroke? A systematic review and narrative synthesis. Int J Behav Nutr Phys Act 2018; 15: 100–100. DOI: 10.1186/s12966-018-0730-0 PMC6192196 30333027

[9] Aguiar LT Nadeau S Martins JC , et al. Efficacy of interventions aimed at improving physical activity in individuals with stroke: a systematic review. Disabil Rehabil 2020; 42: 902–917. 30451539 10.1080/09638288.2018.1511755

[10] Dromerick AW Geed S Barth J , et al. Critical period after stroke study (CPASS): a phase II clinical trial testing an optimal time for motor recovery after stroke in humans. PNAS Nexus 2021; 118: e2026676118. 10.1073/pnas.2026676118 PMC8488696 34544853

[11] Zeiler SR . Should we care about early post-stroke rehabilitation? Not yet, but soon. Curr Neurol Neurosci Rep 2019; 19: 13. 30788609 10.1007/s11910-019-0927-x

[12] Thilarajah S Mentiplay B Bower K , et al. Factors associated with post-stroke physical activity: a systematic review and meta-analysis. Arch Phys Med Rehabil 2018; 99: 1876–1889. 29056502 10.1016/j.apmr.2017.09.117

[13] Preston E Ada L Stanton R , et al. Prediction of independent walking in people who are nonambulatory early after stroke: a systematic review. Stroke 2021; 52: 3217–3224. 34238016 10.1161/STROKEAHA.120.032345

[14] Moher D Liberati A Tetzlaff J , et al. Preferred reporting items for systematic reviews and meta-analyses: the PRISMA statement. Br Med J 2009; 339: b2535. PMC3090117 21603045

[15] Fini NA Simpson D Moore SA , et al. How should we measure physical activity after stroke? An international consensus. Int J Stroke 2023; 18: 1132–1142. 37300499 10.1177/17474930231184108 PMC10614172

[16] Hayden JA van der Windt DA Cartwright JL , et al. Assessing bias in studies of prognostic factors. Ann Intern Med 2013; 158: 280–286. 23420236 10.7326/0003-4819-158-4-201302190-00009

[17] Bonetti D Johnston M . Perceived control predicting the recovery of individual-specific walking behaviours following stroke: testing psychological models and constructs. Br J Health Psychol 2008; 13: 463–478. 17588292 10.1348/135910707X216648

[18] Katz-Leurer M Shochina M . Early cycling test as a predictor of walking performance in stroke patients. Physiother Res Int 2005; 10: –9. 10.1002/pri.19 15991482

[19] Mahendran N Kuys SS Brauer SG . Which impairments, activity limitations and personal factors at hospital discharge predict walking activity across the first 6 months poststroke? Disabil Rehabil 2020; 42: 763–769. 30724628 10.1080/09638288.2018.1508513

[20] Nakao M Izumi S Yokoshima Y , et al. Prediction of life-space mobility in patients with stroke 2 months after discharge from rehabilitation: a retrospective cohort study. Disabil Rehabil 2020; 42: 2035–2042. 30676134 10.1080/09638288.2018.1550533

[21] Park J Shi-Uk L Se Hee J . Prediction of post-stroke functional mobility from the initial assessment of cognitive function. NeuroRehabilitation 2017; 41: 169–177. 28505995 10.3233/NRE-171469

[22] Thilarajah S Bower KJ Pua YH , et al. Modifiable factors associated with poststroke physical activity at discharge from rehabilitation: prospective cohort study. Phys Ther 2020; 100: 818–828. 31995190 10.1093/ptj/pzaa022

[23] Bijleveld-Uitman M van de Port I Kwakkel G . Is gait speed or walking distance a better predictor for community walking after stroke? J Rehabil Med 2013; 45: 535–540. 23584080 10.2340/16501977-1147

[24] Fulk GD He Y Boyne P , et al. Predicting home and community walking activity poststroke. Stroke 2017; 48: 406–411. 28057807 10.1161/STROKEAHA.116.015309

[25] Miller A Pohlig RT Wright T , et al. Beyond physical capacity: factors associated with real-world walking activity after stroke. Arch Phys Med Rehabil 2021; 102: 1880–1887.e1881. 33894218 10.1016/j.apmr.2021.03.023 PMC12323590

[26] Danks KA Pohlig RT Roos M , et al. Relationship between walking capacity, biopsychosocial factors, self-efficacy, and walking activity in persons poststroke. J Neurol Phys Ther 2016; 40: 232–238. 27548750 10.1097/NPT.0000000000000143 PMC5025374

[27] Espernberger KR Fini NA Peiris CL . Personal and social factors that influence physical activity levels in community-dwelling stroke survivors: a systematic review of qualitative literature. Clin Rehabil 2021; 35: 1044–1055. 33586479 10.1177/0269215521993690

[28] Gianella MG Gath CF Bonamico L , et al. Prediction of gait without physical assistance after inpatient rehabilitation in severe subacute stroke subjects. J Stroke Cerebrovasc Dis 2019; 28: 104367. 31519458 10.1016/j.jstrokecerebrovasdis.2019.104367

[29] Kennedy C Bernhardt J Churilov L , et al. Factors associated with time to independent walking recovery post-stroke. J Neurol Neurosurg Psychiatry 2021; 92: 702–708. 33737383 10.1136/jnnp-2020-325125

[30] Hornby TG Henderson CE Holleran CL , et al. Stepwise regression and latent profile analyses of locomotor outcomes poststroke. Stroke 2020; 51: 3074–3082. 32883192 10.1161/STROKEAHA.120.031065 PMC7530096

[31] Mulder M Nijland RH van de Port IG , et al. Prospectively classifying community walkers after stroke: who are they? Arch Phys Med Rehabil 2019; 100: 2113–2118. 31153852 10.1016/j.apmr.2019.04.017

[32] Chu CL Lee TH Chen YP , et al. Recovery of walking ability in stroke patients through postacute care rehabilitation. Biomed J 2023; 46. DOI: 10.1016/j.bj.2022.07.004 PMC10345220 35872227

[33] Covert S Johnson JK Stilphen M , et al. Use of the activity measure for post-acute care “6 clicks” basic mobility inpatient short form and national institutes of health stroke scale to predict hospital discharge disposition after stroke. Phys Ther 2020; 100: 1423–1433. 32494809 10.1093/ptj/pzaa102

[34] Liu Q Jin Y Wang Y , et al. Association between self-efficacy and self-management behaviours among individuals at high risk for stroke: social support acting as a mediator. J Clin Nurs 2023; 32: 71–82. 34981582 10.1111/jocn.16191

[35] Miller A Pohlig RT Reisman DS . Relationships among environmental variables, physical capacity, balance self-efficacy, and real-world walking activity post-stroke. Neurorehabil Neural Repair 2022; 36: 535–544. 35924968 10.1177/15459683221115409 PMC9377718

[36] Morris JH MacGillivray S McFarlane S . Interventions to promote long-term participation in physical activity after stroke: a systematic review of the literature. Arch Phys Med Rehabil 2014; 95: 956–967. 24389402 10.1016/j.apmr.2013.12.016

[37] Stretton CM Mudge S Kayes NM , et al. Interventions to improve real-world walking after stroke: a systematic review and meta-analysis. Clin Rehabil 2017; 31: 310–318. 27056251 10.1177/0269215516640863

[38] Cumming TB Thrift AG Collier JM , et al. Very early mobilization after stroke fast-tracks return to walking: further results from the phase II AVERT randomized controlled trial. Stroke 2011; 42: 153–158. 21148439 10.1161/STROKEAHA.110.594598

[39] Kwah LK Harvey LA Diong J , et al. Models containing age and NIHSS predict recovery of ambulation and upper limb function six months after stroke:an observational study. J Physiother (Elsevier) 2013; 59: 189–197. 10.1016/S1836-9553(13)70183-8 23896334

[40] Olsson OA Persson HC Alt Murphy M , et al. Early prediction of physical activity level 1 year after stroke: a longitudinal cohort study. BMJ Open 2017; 7: e016369. 10.1136/bmjopen-2017-016369 PMC5634455 28780554

[41] Shimizu N Hashidate H Ota T , et al. Daytime physical activity at admission is associated with improvement of gait independence 1 month later in people with subacute stroke: a longitudinal study. Top Stroke Rehabil 2020; 27: 25–32. 31405344 10.1080/10749357.2019.1649916

[42] Botö S Buvarp D Hansson P-O , et al. Physical inactivity after stroke: incidence and early predictors based on 190 individuals in a 1-year follow-up of the fall study of Gothenburg. J Rehabil Med 2021; 53: jrm00224. 10.2340/16501977-2852 PMC8638731 34121128

[43] Pereira S Foley N Salter K , et al. Discharge destination of individuals with severe stroke undergoing rehabilitation: a predictive model. Disabil Rehabil 2014; 36: 727–731. 24654961 10.3109/09638288.2014.902510

[44] Van Criekinge T Heremans C Burridge J , et al. Standardized measurement of balance and mobility post-stroke: consensus-based core recommendations from the third stroke recovery and rehabilitation roundtable. Int J Stroke 2023; 19: 158–168. 37824730 10.1177/17474930231205207

[45] Kwakkel G Lannin NA Borschmann K , et al. Standardized measurement of sensorimotor recovery in stroke trials: consensus-based core recommendations from the stroke recovery and rehabilitation roundtable. Int J Stroke 2017; 12: 451–461. 28697709 10.1177/1747493017711813

[46] Braakhuis HEM Berger MAM Regterschot RGRH , et al. Physical activity dimensions after stroke: patterns and relation with lower limb motor function. J Neuroeng Rehabil 2021; 18: 71. 34895265 10.1186/s12984-021-00960-x PMC8666008

