# Supplementary material for: What factors at discharge predict physical activity and walking outcomes 6 months after stroke? A systematic review
Source: Clin Rehabil. 2024 Jul 25;38(10):1393–403. doi: 10.1177/02692155241261698 (PMC11528935; doi:10.1177/02692155241261698)
Supplement: sj-docx-1-cre-10.1177_02692155241261698 - Supplemental material for What factors at discharge predict physical activity and walking outcomes 6 months after stroke? A systematic review [file sj-docx-1-cre-10.1177_02692155241261698.docx]

**Supplementary material 1: Search strategy**

1. Database: CINAHL

Keyword search string:

((Stroke OR "Cerebrovascular accident" OR CVA OR "brain hemorrhage" OR "brain attack")) AND ((walking OR ambulation OR locomotion) AND (physical activity OR physical fitness OR physical exercise))

Subject headings search string:

(((MH "Stroke+") OR (MH "Stroke Patients")) AND (MH "Walking+") AND (MH "Leisure activities+") AND (MH "Exercise+"))

1. Database: PubMed

Keyword search string:

((Stroke OR Cerebrovascular accident OR CVA OR brain hemorrhage OR brain attack) AND (walking* OR walkers* OR ambulation*) AND (exercise* OR physical activity* OR physical fitness*))

PubMed Mesh and keyword search string:

(("Stroke"[Mesh] OR "Cerebrovascular accident" OR CVA OR "brain hemorrhage" OR "brain attack") AND (("Exercise"[Mesh] OR "physical activity" OR "physical fitness") OR (walking OR walkers OR ambulation)))

1. Scopus search string

(TITLE-ABS-KEY(stroke OR {Cerebrovascular accident} OR cva OR {brain hemorrhage} OR {brain attack}) AND (TITLE-ABS-KEY({walking} OR {walkers} OR {ambulation}) OR TITLE-ABS-KEY({physical activity} OR {physical fitness} OR {exercise})))

1. Web of Science search string

TS=(Stroke OR "Cerebrovascular accident" OR CVA OR "brain hemorrhage" OR "brain attack") AND TS=("physical activity" OR "physical fitness" OR "exercise") AND ALL=("walking" OR "walkers" OR "ambulation") AND ALL=("recovery" OR "outcome" OR "success")
